# Prognostic Biomarkers for Pancreatic Ductal Adenocarcinoma: An Umbrella Review

**DOI:** 10.3389/fonc.2020.01466

**Published:** 2020-09-17

**Authors:** Yizhi Wang, Xi Zhong, Li Zhou, Jun Lu, Bolun Jiang, Chengxi Liu, Junchao Guo

**Affiliations:** ^1^Department of General Surgery, Peking Union Medical College Hospital, Chinese Academy of Medical Sciences/Peking Union Medical College, Beijing, China; ^2^Key Laboratory of Precision Diagnosis and Treatment of Gastrointestinal Tumors, Department of Surgical Oncology and General Surgery, Ministry of Education, The First Affiliated Hospital of China Medical University, Shenyang, China

**Keywords:** pancreatic ductal adenocarcinoma, biomarkers, umbrella review, prognosis, survival

## Abstract

**Background:** Pancreatic ductal adenocarcinoma (PDAC) leads to the majority of cancer-related deaths due to its morbidity with similar mortality. Lack of effective prognostic biomarkers are the main reason for belated post-operative intervention of recurrence which causes high mortality. Numerous systematic reviews and meta-analyses have explored the prognostic value of biomarkers in PDAC so far. In this article, we performed an umbrella review analyzing these studies to provide an overview of associations between prognostic biomarkers and PDAC survival outcome and synthesized these results to guide better clinical practice.

**Methods:** Systematic reviews and meta-analyses investigating the associations between PDAC survival outcomes and prognostic biomarkers were acquired via the PubMed and Embase databases from inception till February 1, 2020. Associations supported by nominally statistically significant results were classified into strong, highly suggestive, suggestive, and weak based on several critical factors such as the statistical significance of summary estimates, the number of events, the estimate of the largest study included, interstudy heterogeneity, small-study effects, 95% predictive interval (PI), excess significance bias, and the results of credibility ceiling sensitivity analyses.

**Results:** We included 41 meta-analyses containing 63 associations between PDAC survival outcomes and prognostic biomarkers. Although, none was supported by strong evidence among these associations, an association between C-reactive protein to albumin ratio (CAR) and PDAC overall survival (OS) and an association between neutrophil-lymphocyte ratio (NLR) and PDAC OS were supported by highly suggestive evidence. Otherwise, the association between lactate dehydrogenase (LDH) and PDAC OS was supported by suggestive evidence. The remaining 60 associations were supported by weak or not suggestive evidence.

**Conclusion:** Associations between CAR or NLR and PDAC OS were supported by highly suggestive evidence. And the association between LDH and PDAC OS was supported by suggestive evidence. Although the methodological quality of the included systematic reviews and meta-analyses which were evaluated by AMSTAR2.0 is generally poor, the identification of the relatively robust prognostic biomarkers of PDAC may guide better post-operative intervention and follow-up to prolong patients' survival.

## Introduction

Pancreatic ductal adenocarcinoma (PDAC) leads to major cancer-related death due to its morbidity with similar mortality. The mortality remains unchanged and may even have increased during the last few decades (1). The current estimated 5-year overall survival (OS) of PDAC is <8% and PDAC ranks fourth among cancer-related deaths in the US both in men and women for consecutive years, and the 5-year OS reduces to a dismal 3% when distant metastases occurs (2–5). In China, PDAC ranks sixth among the most lethal cancers and an increasing number of younger patients have been subjected to this disease (6). To raise the rate of survival in PDAC patients, more financial costs have been made around the world (7, 8). Currently, surgical treatment is still the only radical therapy for PDAC (9, 10). Although adjuvant chemotherapy may prolong the survival of PDAC patients, the prognosis of PDAC patients remains poor due to the lack of early diagnostic methods and accurate recurrence and prognostic biomarkers (11–13).

Biomarkers are biological molecules or molecular combinations involved in tumor progression by modulating various signaling pathways which can be used in early diagnosis and prognostic evaluation for cancers (14, 15). Detecting efficient prognostic biomarkers preoperatively can ensure a correct and individual evaluation of the OS and recurrence potential. By doing this, clinical practitioners can perform specific follow-up strategies and timely intervention of possible recurrence to prolong patients' 5-year OS and decrease the mortality (16). Of note, the most frequently evaluated serum biomarker for prognostic evaluation after operation is carbohydrate antigen 19-9 (CA19-9) for PDAC (17, 18). It was reported that CA19-9 could precede radiological evidence for about 3–6 months predicting tumor recurrence (19). However, CA19-9 may not be so effective or accurate in prognostic evaluation. Other serum biomarkers have been proven to have better sensitivity and specificity than CA19-9 in PDAC but without widespread clinical application, such as circulating tumor DNA, miRNAs, lncRNAs, and even circRNAs (20–23). There have been numerous systematic reviews and meta-analyses focusing on prognostic biomarkers of PDAC so far. However, to the best of our knowledge, the results of these systematic reviews and meta-analyses have not been synthetically evaluated. To provide an overview of associations between PDAC survival outcomes and prognostic biomarkers, and to find robust prognostic biomarkers to guide clinical practice, an umbrella review analyzing currently available meta-analyses and systematic reviews and rating the evidence depending on the credibility of these associations was performed.

## Methods

### Literature Retrieval and Eligibility Criteria

A literature search of the PubMed and Embase databases was conducted for systematic reviews and meta-analyses investigating the associations between PDAC survival outcomes and prognostic biomarkers independently by two experienced researchers (YW and XZ) from inception till February 1, 2020. No filters regarding language or publication time were applied. The “related function” was used to include more articles. Then manual retrieval from the citations of included studies was also applied to supplement the potentially missing literature. The relevant search terms are presented below: (pancreatic cancer OR PDAC OR pancreatic ductal adenocarcinoma OR pancreatic carcinoma OR pancreatic neoplasm) AND (biomarkers OR prognosis OR prognostic biomarkers) AND (systematic reviews OR meta-analyses). The titles and abstracts of these studies were independently browsed by the same two experienced reviewers (YW and XZ). Then full texts of the potentially relevant ones were carefully read by both reviewers. The studies which met the inclusion criteria were finally included in the umbrella review.

### Inclusion and Exclusion Criteria

The inclusive and exclusive criterion of the evaluated meta-analyses was shown below: (1) Studies including associations between prognostic biomarkers, rather than diagnostic or pharmacodynamic biomarkers, and PDAC survival outcomes such as overall survival (OS), progression-free survival (PFS), disease-free survival (DFS), and cancer-specific survival (CSS) were included. (2) Studies investigating PDAC risk factors and genetic polymorphism and studies focusing on benign pancreatic lesions, such as solid pseudopapillary tumors of the pancreas, pancreatic cystic tumors, and intraductal papillary mucinous neoplasm were excluded. (3) Meta-analyses containing just one original study or not providing sufficient data were excluded in our present review. When two or more meta-analyses discussed the same association, we only included the meta-analyses with the latest or the largest primary studies.

### Data Extraction

Two investigators (YW and XZ) independently extracted the data from included meta-analyses and contradictions between the two reviewers were resolved through discussion with a third reviewer (JG). The name of first author, country, biomarker name, cases number, population size, and relative risk estimation, including risk ratio (RR), odds ratio (OR), and hazard ratio (HR), and the corresponding 95% confidential interval (CI) were retrieved from each of the included meta-analysis.

### Quality Assessment

AMSTAR (A MeaSurement Tool to Assess Systematic Reviews) version 2.0 was used to evaluate the methodological quality of each of the included systematic reviews (24). AMSTAR is an important tool used in umbrella reviews to conduct methodological quality assessment of the included systematic reviews and meta-analyses. AMSTAR version 2.0 is an updated version of AMSTAR and contains 16 items to create a more comprehensive and rational evidence evaluation. According to the comprehensive evaluation of the 16 items, the methodological quality of the included studies can be graded as high, moderate, low, or critically low rather than obtaining an overall score.

### Statistical Analysis

Random-effect models were used to evaluate the synthesized summary effects for the included meta-analyses. The summary RR estimates, the 95% CI, and the corresponding *P-*values were calculated. *P* < 0.05 was deemed as significant.

Cochran's *Q*-test and *I*^2^ statistic were applied to assess interstudy heterogeneity. And *I*^2^ > 50% indicates significant heterogeneity (25). Ninety-five percent prediction interval (95% PI), which predicts the likely effect in an individual setting rather than just an average effect across all included studies in a meta-analysis, was used to facilitate the application of the results to clinical practice (26, 27).

Small-study effects were assessed using Egger's regression asymmetry test. Small-study effects were considered detected when a *P* < 0.1 was reached (27).

Potential reporting selection biases or publication biases can be detected by the excess significance test. A Chi-square test with a two-tailed *P* < 0.1 as the statistical significance threshold was used to assess whether the actual observed number (O) was different from the expected number (E) (28). The E number which was the sum of the statistical power estimates of the component studies in the included meta-analyses expecting to be statistically significant was calculated using an algorithm of a non-central t distribution with the relative risk estimate of the largest study set as the plausible effect size. The excess significance test was considered positive in cases where both O>E and *P* < 0.1.

Credibility ceiling sensitivity analyses were performed to account for the inherent methodological limits of observational studies. The level of the credibility ceiling was set at 10% for the present study which indicates that the weight of an observational study in the summary effect is limited to 10% no matter how well the study was conducted (29).

### Strength of Existing Evidence

Based on the results of a series of aforementioned analyses, the strength of the statistically significant (*P* < 0.05) associations between risk factors and risk of PDAC can be categorized into four levels. To reach the “strong evidence” level, the included meta-analysis were expected to show a *P*-value of random-effect model smaller than 10^−6^, include more than 1000 PDAC cases, the 95% PI should exclude the null value, have a smaller heterogeneity with *I*^2^ < 50%, have no evidence of small-study effect, and an excess significance bias and also survive the 10% credibility ceiling test. “Highly suggestive” evidences refer to associations with a *P*-value of random-effect model smaller than 10^−6^ and covering more than 1,000 cases. A “suggestive” association was required to reach a *P* < 10^−3^ and include more than 1,000 cases. The rest of the associations where *P*-value was statistically significant were graded as “weak” evidence (30, 31).

## Results

### Characteristics of the Included Systematic Reviews and Meta-Analyses

**Figure 1:**
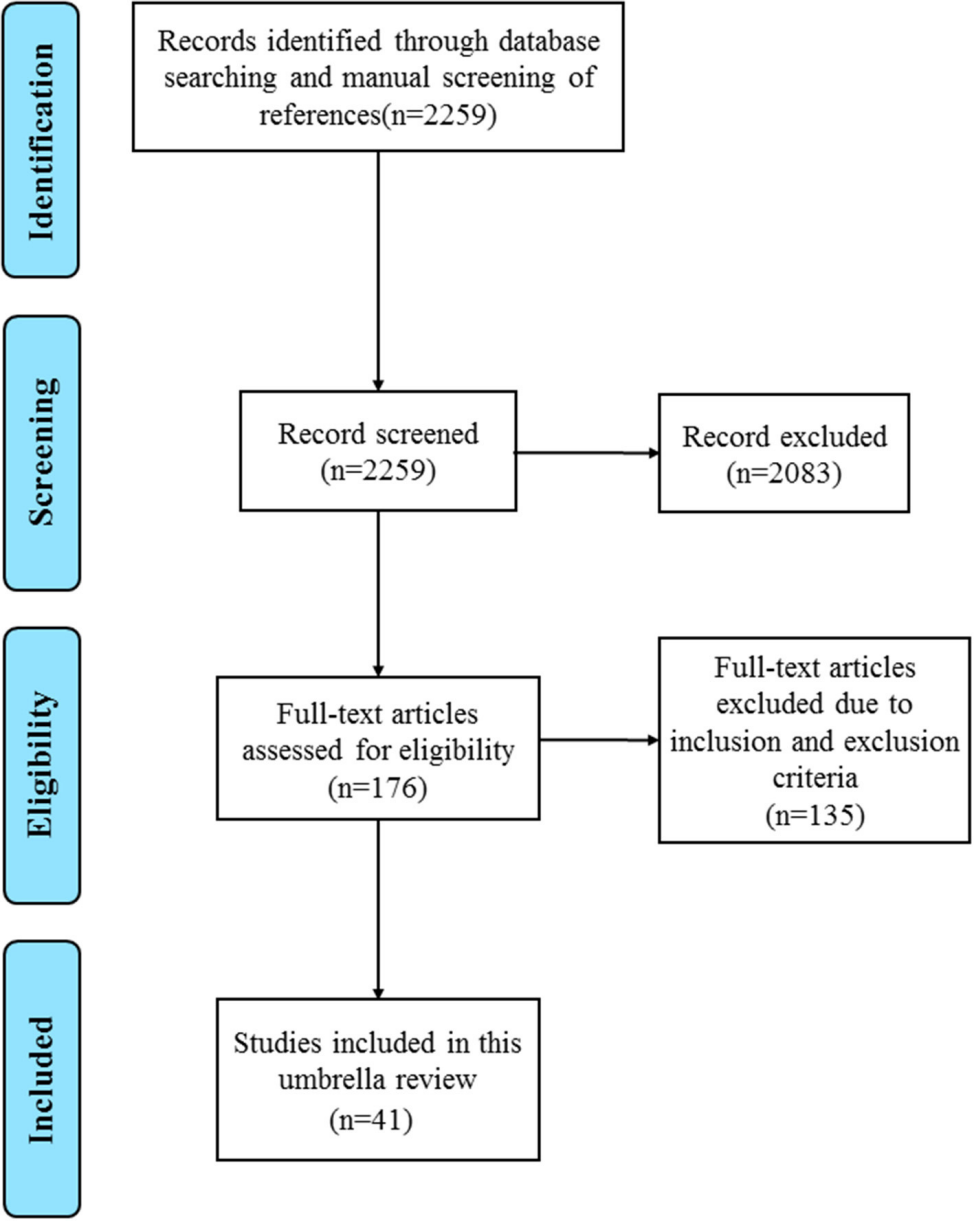
The flow diagram of the study selection.

In total, 2259 records were extracted from the literature search and manual screening using the PubMed and Embase databases. A total of 2,083 records were excluded due to an irrelevant theme after browsing titles and abstracts from the acquired records. Finally, 41 of the remaining 176 studies which met the inclusion criteria were included in the present umbrella review after a full-text review (32–72). The search flowchart is shown in Figure 1. And Supplementary Table 1 displays the full list of 176 meta-analyses and the exclusive reasons for the 135 studies. A total of 63 different associations between PDAC survival outcomes and prognostic biomarkers were covered in the included 41 meta-analyses which contained more than 31,000 subjects and over 300 primary studies. Characteristics of the 63 associations in the included meta-analyses were presented in Table 1. Data of the included primary studies in the 41 meta-analyses were extracted, processed, and coded in order to conduct further analysis.

**Table 1:** Characteristics of the 63 associations in the included 41 systematic reviews and meta-analyses.

**References**	**Biomarker**	**Association between biomarker and pancreatic cancer**	**Effect metrics**	**Country**	**No. of study estimates**	**No. of cases/total population**	**Summary relative risk estimate (95%CI)**
Rezende et al. (30)	AEG-1	OS	HR	China	2	82^#^/194	2.41 (1.63–3.57)
Markozannes et al. (31)	B7-H4	OS	HR	China	5	349/372	3.00 (2.20–4.10)
Luo et al. (32)	Bax	OS	HR	UK	5	185^#^/274	0.63 (0.48–0.83)
	Bcl-2	OS	HR	UK	5	267/314	0.51 (0.38–0.68)
	EGFR	OS	HR	UK	4	178^#^/250	1.35 (0.80–2.27)
	P16	OS	HR	UK	3	28^#^/229	0.63 (0.43–0.92)
	P53	OS	HR	UK	17	510^#^/933	1.22 (0.96–1.56)
	VEGF	OS	HR	UK	11	537^#^/767	1.51 (1.81–1.92)
Chen et al. (33)	CAR	OS	HR	China	11	1,666/1934	1.86 (1.53–2.26)
Smith et al. (34)	CD133	5-year OS	HR	China	11	552^#^/723	1.22 (0.98–1.52)
Fu et al. (35)	CD44	5-year OS	OR	China	5	284/318	0.52 (0.30–0.91)
Li et al. (36)	CXCL12	OS	HR	UK	4	354/439	1.54 (1.21–1.97)
		PFS	HR	UK	2	84/104	1.79 (1.05–3.04)
Liu et al. (37)	CXCR4	OS	HR	China	8	1,131/1262	1.27 (1.00–1.61)
Samarendra et al. (38)	CXCR7	OS	HR	China	2	503/584	2.72 (1.11–6.66)
Ding et al. (39)	COX-2	OS	HR	China	6	498^#^/712	1.48 (1.12–1.85)
Fan et al. (40)	DTCs/CTCs	OS	HR	Australia	12	561^#^/829	1.84 (1.37–2.45)
		PFS	HR	Australia	4	272/292	1.93 (1.19–3.11)
Wang et al. (41)	DLL4	OS	HR	China	2	105/125	2.13 (1.37–2.32)
	Notch3	OS	HR	China	3	53^#^/166	2.05 (1.49–2.82)
Stephenson et al. (42)	E-cadherin	OS	HR	UK	2	172/197	1.58 (1.22–2.22)
	Ki-67	OS	HR	UK	4	196/252	2.42 (1.87–3.14)
	pAkt	OS	HR	UK	2	74/104	0.59 (0.33–1.07)
	P21	OS	HR	UK	3	154/163	0.49 (0.33–0.73)
	P27	OS	HR	UK	5	309/356	1.11 (0.84–1.47)
	Survivin	OS	HR	UK	2	105/114	0.46 (0.29–0.73)
	TP	OS	HR	UK	2	120/144	2.03 (1.22–3.38)
	TS	OS	HR	UK	2	176/204	1.05 (0.73–1.51)
Ye et al. (43)	FGFR2	3-year OS	OR	China	3	135/177	1.66 (0.42–6.53)
		5-year OS	OR	China	3	148/177	1.04 (0.37–2.91)
Jamieson et al. (44)	FoxM1	OS	HR	China	3	237/266	1.73 (1.05–2.86)
Liu et al. (45)	GLUT1	OS	HR	China	8	451/538	1.79 (1.19–2.70)
Dai et al. (46)	HDAC1	OS	HR	China	4	208/244	1.43 (0.71–2.88)
Sharen et al. (47)	hENT1	OS	HR	UK	7	679/770	0.52 (0.38–0.72)
		DFS	HR	UK	6	313^#^/536	0.58 (0.42–0.79)
Cao et al. (48)	HER2	OS	HR	China	4	132^#^/676	1.87 (0.64–5.46)
Bird et al. (49)	HIF-1α	OS	HR	China	6	262^#^/422	1.88 (1.39–2.56)
Li et al. (50)	HIF-2α	OS	HR	China	3	411/443	1.97 (1.42–2.75)
Ye et al. (51)	HK2	OS	HR	China	3	110^#^/235	1.11 (0.58–2.11)
Luo et al. (52)	HMGB1	OS	HR	China	2	101/123	2.61 (1.48–4.59)
Wu et al. (53)	LDH	OS	HR	China	18	3,137/3345	1.58 (1.31–1.91)
Wu et al. (54)	LMR	OS	HR	China	5	668/748	0.59 (0.41–0.85)
Gan et al. (55)	LncRNA loc285194	OS	HR	Iran	2	108/179	1.97 (1.05–3.68)
Mao et al. (56)	LncRNA UCA1	OS	HR	China	2	160/208	1.58 (1.01–2.15)
Mehrad-Majd et al. (57)	L1CAM	OS	HR	China	3	257/311	0.96 (0.42–2.21)
Liu et al. (58)	MiRNA-203	OS	HR	China	3	142^#^/295	1.19 (1.09–1.31)
Hua et al. (59)	NLR	OS	HR	China	43	6,479^#^/8252	1.81 (1.59–2.05)
		DFS	HR	China	8	1,141/1236	1.66 (1.17–2.35)
Shao et al. (60)	PD-L1	OS	HR	China	8	766/912	1.63 (1.34–1.98)
		CSS	HR	China	3	381/490	1.86 (1.34–2.57)
Zhou et al. (61)	Plasma fibrinogen	OS	HR	China	6	601/800	1.56 (1.13–2.15)
Hu et al. (62)	PLR	OS	HR	China	3	591/632	1.00 (0.92–1.09)
Ji et al. (63)	PKM2	OS	HR	China	4	259/313	1.41 (0.68–2.93)
Yin et al. (64)	Podoplanin^+^fbroblast	OS	HR	China	3	184/231	2.20 (1.40–3.46)
		DFS	HR	China	2	165/200	1.97 (1.37–2.84)
Zhu et al. (65)	RKIP	OS	HR	China	3	256/276	0.76 (0.51–1.01)
		DFS	HR	China	3	237/276	0.71 (0.28–1.13)
Hu et al. (66)	RRM1	OS	HR	China	9	666/733	1.56 (0.95–2.17)
		DFS	HR	China	3	185/220	1.37 (0.25–2.48)
Yu et al. (67)	Smad4	OS	HR	China	10	1,446/1734	0.61 (0.38–0.99)
Zhang et al. (68)	SPARC	OS	HR	China	7	908/1128	1.55 (1.11–2.17)
Wang et al. (69)	STAT3	5-year OS	OR	China	3	17/243	9.71 (1.80–52.41)
Han et al. (70)	ZEB1	OS	HR	China	2	120/188	1.49 (1.07–2.06)

# *Contain missing values; CI, confidence interval; OS, overall survival; CSS, cancer-specific survival; DFS, disease free survival; PFS, progression free survival; AEG-1, astrocyte elevated gene-1; CAR, C-reactive protein to albumin ratio; COX-2, cyclooxygenase-2; DLL4, delta like ligand 4; DTCs/CTCs, disseminated tumor cells/circulating tumor cells; EGFR, epidermal growth factor receptor; VEGF, vascular endothelial growth factor; FGFR2, fibroblast growth factor receptors; FoxM1, Forkhead Box M1; GLUT1, glucose transporter type 1; HDAC1, histone deacetylase 1; hENT1, human equilibrative nucleoside transporter 1; HER2, human epidermal growth factor receptor-2; HIF-1α, hypoxia inducible factor-1α; HIF-2α, hypoxia inducible factor-2α; HK2, hexokinase 2; HMGB1, high mobility group box 1; LDH, lactate dehydrogenase; LMR, lymphocyte-to-monocyte ratio; LncRNA, long non-coding RNA; L1CAM, L1 cell adhesion molecule; MiRNA, MicroRNA; NLR, neutrophil-to-lymphocyte ratio; pAkt, phosphorylated protein kinase B; PD-L1, programmed cell death ligand 1; PLR, platelet-to-lymphocyte ratio; PKM2, pyruvate kinase M2; RKIP, Raf kinase inhibitor protein; RRM1, ribonucleotide reductase M1; SPARC, secreted protein acidic and rich in cysteine; STAT3, signal transducer and activator of transcription proteins 3; TS, thymidylate synthase; TP, thymidylate phosphorylase; VEGF, vascular endothelial growth factor; ZEB1, zinc finger E-box binding homeobox 1*.

### Quality Assessment Methodology Using AMSTAR 2.0

The 16-item AMSTAR 2.0 tool was recruited to assess the methodological quality of the 41 included meta-analyses. The results showed that qualities of all the included studies were considered as critically low. All of these meta-analyses had more than two critical flaws [usually in items 2 (41/41,100%), 7 (41/41,100%), and 13 (41/41,100%)] and several non-critical flaws [usually in items 3 (41/41,100%), 10 (41/41,100%), and 12 (41/41,100%)]. It is worth noting that studies with more than one critical flaw were considered as critically low-quality studies regardless of non-critical flaws. Considering the critically low quality of all the included systematic reviews, the results should be interpreted cautiously. The detailed results and rating criteria are shown in Supplementary Table 2.

### Summary Effect Size

The quantitative syntheses of the 63 associations were conducted using a random-effect model to provide more conservative estimates. A total of 44 of the 63 associations in the included meta-analyses were statistically significant with *P* < 0.05, while the remaining 19 associations showing *P* > 0.05 (Table 2 and Supplementary Table 3). Of the statistically significant associations, four reached *P* < 10^−6^, namely, associations between C-reactive protein to albumin ratio (CAR), neutrophil-lymphocyte ratio (NLR), or B7-H4 and PDAC OS and the association between CD133 and 5-year OS of PDAC. And 20 other associations reached moderate statistical significance (*P* < 10^−3^). More than half of the associations that reached statistical significance reported an increased risk of mortality of PDAC, indicating a potential biomarker role in PDAC prognostic prediction. However, the remaining statistically significant associations reported a decreased risk of mortality of PDAC, such as B7-H4 and PDAC OS, and Bax and PDAC OS.

**Table 2:** Evidence-rating results based on the results of statistical analyses of the 63 associations.

**Study**	**Association between biomarkers and pancreatic cancer**	Summary relative risk estimate (random-effect *P*)^*^	**Cases >1,000**	**Largest study relative risk estimate *P* < 0.05**	***I*^**2**^ < 50%**	**Small study effects**	**95% prediction interval exclude the null value**	**Excess significance**	**10% credibility ceiling survival**
**Associations supported by highly suggestive evidence** **(****2****)**									
Chen et al. (33)	CAR OS	+++	+	+	–	–	+	+	+
Hua et al. (59)	NLR OS	+++	+	+	–	+	–	+	+
**Associations supported by suggestive evidence** **(****1****)**									
Wu et al. (53)	LDH OS	++	+	–	–	–	–	+	+
**Associations supported by weak evidence** **(****41****)**									
Rezende et al. (30)	AEG-1 OS	++	–	+	+	NA	NA	–	–
Markozannes et al. (31)	B7-H4 OS	+++	–	+	+	–	+	+	+
Luo et al. (32)	Bax OS	++	–	+	+	–	+	+	+
	Bcl-2 OS	++	–	+	+	–	+	+	+
	P16 OS	+	–	–	+	+	–	–	–
	VEGF OS	++	–	+	–	–	–	–	+
Smith et al. (34)	CD133 5-year OS	+++	–	+	+	+	+	+	+
Fu et al. (35)	CD44 5-year OS	+	–	–	+	–	–	–	–
Li et al. (36)	CXCL12s OS	++	–	–	+	–	–	–	+
	CXCL12s PFS	+	–	+	+	NA	NA	–	–
Liu et al. (37)	CXCR4 PFS	+	+	–	+	+	–	–	–
Samarendra et al. (38)	CXCR7 OS	+	–	–	+	–	–	–	–
Ding and Du (39)	COX-2 OS	++	–	+	+	–	+	–	+
Fan et al. (40)	DTCs/CTCs OS	++	–	–	+	–	–	+	+
	DTCs/CTCs PFS	+	–	–	–	–	–	+	+
Wang et al. (41)	DLL4 OS	++	–	+	+	NA	NA	–	–
	Notch3 OS	++	–	+	+	+	–	+	+
Stephenson et al. (42)	E-cadherin OS	+	–	+	+	NA	NA	–	–
	Ki-67 OS	+	–	+	–	–	–	–	+
	P21 OS	+	–	+	–	–	–	+	–
	TP OS	+	–	+	+	NA	NA	–	–
Liu et al. (45)	GLUT1OS	+	–	–	–	+	–	+	–
Sharen et al. (47)	hENT1 OS	++	–	+	–	–	–	+	+
	hENT1 DFS	++	–	+	–	+	–	+	–
Bird et al. (49)	HIF-1α OS	++	–	+	+	–	+	–	+
Li et al. (50)	HIF-2α OS	++-	–	+	+	–	–	+	+
Luo et al. (52)	HMGB1 OS	++-	–	+	+	NA	NA	+	–
Wu et al. (54)	LMR OS	+	–	+	–	–	–	+	–
Gan et al. (55)	LncRNA loc285194 OS	+	–	+	+	NA	NA	–	–
Mao et al. (56)	LncRNA UCA1 OS	+	–	+	+	NA	NA	+	–
Hua et al. (59)	NLR DFS	+	+	+	–	–	–	+	–
Shao et al. (60)	PD-L1 OS	++	–	+	+	–	+	+	+
	PD-L1 CSS	++	–	+	+	–	–	–	+
Zhou et al. (61)	Plasma fibrinogen OS	+	–	+	–	–	–	+	–
Yin et al. (64)	Podoplanin^+^fbroblast OS	++	–	+	+	–	–	–	–
	Podoplanin^+^fbroblast DFS	++	–	+	+	NA	NA	+	–
Hu et al. (66)	RRM1 DFS	++	–	+	–	–	–	+	+
Yu et al. (67)	Smad4 OS	+	+	+	–	–	–	–	+
Zhang et al. (68)	SPARC OS	+	–	–	–	–	–	+	–
Wang et al. (69)	STAT3 5-year OS	+	–	–	+	–	–	–	+
Han et al. (70)	ZEB1 OS	+	–	–	+	NA	NA	–	–
**Associations supported by not suggestive evidence** **(****19****)**									
Luo et al. (32)	EGFR OS	–	–	–	–	–	–	–	–
	P53 OS	–	–	–	–	–	–	–	–
Ye et al. (43)	FGFR2 3-year OS	–	–	+	–	–	–	–	+
	FGFR2 5-year OS	–	–	–	+	–	–	–	–
Jamieson et al. (44)	FoxM1 OS	–	–	–	+	–	–	+	–
Dai et al. (46)	HIDAC1 OS	–	–	–	–	–	–	+	–
Cao et al. (48)	HER2 OS	–	–	–	–	+	–	–	–
Ye et al. (51)	HK2 OS	–	–	+	–	–	–	–	–
Mehrad-Majd et al. (57)	LICAM OS	–	–	–	–	–	–	+	–
Liu et al. (58)	MiRNA-203 OS	–	–	+	+	–	–	–	–
Stephenson et al. (42)	pAkt OS	–	–	+	–	NA	NA	–	–
	P27 OS	–	–	–	–	–	–	+	–
	Survivin OS	–	–	+	–	NA	NA	+	–
	Thymidylate synthase OS	–	–	+	–	NA	NA	+	–
Hu et al. (62)	PLR OS	–	–	–	+	–	–	–	–
Ji et al. (63)	PKM2 OS	–	–	+	–	–	–	+	–
Zhu et al. (65)	RKIP OS	–	–	–	+	–	–	+	–
	RKIP DFS	–	–	–	–	–	–	+	–
Hu et al. (66)	RRM1 DFS	–	–	+	–	–	–	+	–

* *P-value calculated using random-effect model: ^+++^P < 10^−6^; ^++^P < 10^−3^; ^+^P < 0.05; ^−^P > 0.05. For other items, +, yes; –, no*.

*CI, confidence interval; NA, not available for meta-analysis that included less than three studies; OS, overall survival; CSS, cancer-specific survival; DFS, disease free survival; PFS, progression free survival; AEG-1, astrocyte elevated gene-1; CAR, C-reactive protein to albumin ratio; COX-2, cyclooxygenase-2; DLL4, delta like ligand 4; DTCs/CTCs, disseminated tumor cells/circulating tumor cells; EGFR, epidermal growth factor receptor; VEGF, vascular endothelial growth factor; FGFR2, fibroblast growth factor receptors; FoxM1, Forkhead Box M1; GLUT1, glucose transporter type 1; HDAC1, histone deacetylase 1; hENT1, human equilibrative nucleoside transporter 1; HER2, human epidermal growth factor receptor-2; HIF-1α, hypoxia inducible factor-1α; HIF-2α, hypoxia inducible factor-2α; HK2, hexokinase 2; HMGB1, high mobility group box 1; LDH, lactate dehydrogenase; LMR, lymphocyte-to-monocyte ratio; LncRNA, long non-coding RNA; L1CAM, L1 cell adhesion molecule; MiRNA, MicroRNA; NLR, neutrophil-to-lymphocyte ratio; pAkt, phosphorylated protein kinase B; PD-L1, programmed cell death ligand 1; PLR, platelet-to-lymphocyte ratio; PKM2, pyruvate kinase M2; RKIP, Raf kinase inhibitor protein; RRM1, ribonucleotide reductase M1; SPARC, secreted protein acidic and rich in cysteine; STAT3, signal transducer and activator of transcription proteins 3; TS, thymidylate synthase; TP, thymidylate phosphorylase; VEGF, vascular endothelial growth factor; ZEB1, Zinc finger E-box binding homeobox 1*.

### Heterogeneity

Of the 63 associations, there were 17 associations with moderate to high heterogeneity (*I*^2^ = 50–75%) and 13 associations with high heterogeneity (*I*^2^ > 75%). When we further evaluated the interstudy heterogeneity using 95% PI estimates, we found eight associations with the null value excluded (Table 2 and Supplementary Table 3).

### Small-Study Effects

Among the 63 associations between PDAC survival outcomes and prognostic biomarkers, small-study effects were detected in eight associations [NLR OS, P16 OS, CD133 5-year OS, CXCR4 PFS, Notch3 OS, glucose transporter type 1(GLUT1) OS, human equilibrative nucleoside transporter 1 (hENT1) DFS, and Human epidermal growth factor receptor-2 (HER2) OS] according to the Egger's test (*P* < 0.1) as shown in Table 2 and Supplementary Table 3.

### Excess Significance

Significant excess significance (O>E and *P* < 0.1) was shown in 35 of the 63 associations (Table 2 and Supplementary Table 3).

### 10% Credibility Ceiling

There were 37 of the 63 associations that survived the 10% credibility ceiling (*P* < 0.1) which included all associations graded as highly suggestive, or suggestive and most of the associations categorized as weak evidence. Table 2 and Supplementary Table 3 show the detailed information.

### Robustness of Evidence

Among the 63 associations between PDAC survival outcomes and prognostic biomarkers, none was supported by strong evidence. However, associations between CAR or NLR and PDAC OS were supported by highly suggestive evidence. Besides, association between lactate dehydrogenase (LDH) and PDAC OS. The remaining 60 associations were supported by weak or not suggestive evidence. Detailed results of these analysis processes are shown in Supplementary Table 3.

## Discussion

### Principal Finding and Existing Evidence Interpretation

Both diagnostic and prognostic biomarkers are vital to PDAC treatment and can prolong the survival of PDAC patients. However, it is difficult to find valuable diagnostic biomarkers of PDAC due to its insidious onset and retroperitoneal localized lesions. Therefore, in this umbrella review, we mainly focused on prognostic biomarkers evaluation in order to guide clinical practice and the timely detection of early post-operative recurrence to prolong the survival of the patients (73).

To the best of our knowledge, the present umbrella review is the first to comprehensively collect the existing and available meta-analyses and assess the robustness of evidence to provide an overview of associations between PDAC survival outcomes and prognostic biomarkers. Generally, 41 meta-analyses containing 54 different prognostic biomarkers which consisted of both tissue biomarkers and serum biomarkers were included in this umbrella review. None of the associations were supported by strong evidence. However, two associations were supported by highly suggestive evidence, namely, an association between CAR and PDAC OS and an association between NLR and PDAC OS. Only one association was supported by suggestive evidence, an association between LDH and PDAC OS. And the remaining 60 associations were supported by either weak or not suggestive evidence. Nevertheless, the results should be cautiously interpreted given the relatively poor quality and small number of samples of the meta-analyses included in our umbrella review evaluated by AMSTAR2.0.

### Comparison With Other Studies and Possible Explanations

In our study, some inflammation-based prognostic biomarkers acquired stronger evidence grading rather than tissue biomarkers. For example, associations between CAR or NLR and PDAC OS were supported by highly suggestive evidence. PDAC can be a deeline in its advanced stage, even at a local advanced stage, which can cause cachexia of patients leading to high mortality (74). Systemic inflammation response (SIR) seems to play an important role in cachexia, such as weight loss and functional decline (75, 76). Elevated C-reactive protein (CRP) is one of the most frequently used biomarkers indicating systemic inflammation and extreme hypoalbuminemia is common in advanced PDAC patients with refractory cachexia due to severe malnutrition. According to what we have mentioned above, preoperative level of CAR implies the systemic inflammation extent of patients, and a higher preoperative CAR level may relate to more severe cachexia and a poorer prognosis in patients. Actually, several studies have indicated the prognostic value of CAR in PDAC. A study by Ikuta et al. indicated that in 136 PDAC patients, preoperative CAR was an independent poor OS predictor using multivariate analysis. Moreover, the prognostic evaluation power of CAR was significantly higher than other inflammation-based factors such as NLR, platelet to lymphocyte ratio (PLR), and lymphocyte to monocyte ratio (LMR) (77). Besides, the study by Arima et al. also confirmed this result through using CAR at day 14 after an operation (78). NLR is the other prognostic biomarker supported by highly suggestive evidence. In the process of SIR, the level of neutrophil increases to secrete various cytokines. As SIR mitigates, the level of NLR decreases which may explain why NLR can be used as a prognostic biomarker in PDAC patients. Several studies suggested that the prognostic accuracy of the combination of post-operative NLR and AJCC 8th edition is much better than that of the combination of the TNM staging system and AJCC 8th edition (79, 80). Moreover, a recent study unveiled that preoperative NLR may also be a useful prognostic biomarker in PDAC patients undergoing surgical resection (79).

The association between LDH and PDAC OS was supported by suggestive evidence in our umbrella review. LDH is a vital enzyme in glycolysis which can facilitate the conversion of pyruvate to lactate. And the serum LDH level is higher in patients than in healthy individuals which indicates a tumor-promoting role (81). Therefore, LDH may play an important role in tumor progression, especially in PDAC whose microenvironment is usually hypoxic. A meta-analysis showed that high level of glycolysis-related proteins including LDH5 were associated with shorter OS of various cancer patients including PDAC patients (82). Moreover, LDH combined with other biomarkers can also be prognostic in PDAC. For instance, the ratio of LDH to albumin (LAR) can be a prognostic indicator for unresectable PDAC patients. Besides, the combination of LAR, CEA, and the TNM staging system can improve prognosis predictive power compared with the TNM staging system alone (83).

Several studies have revealed the prognostic role of CA19-9 in PDAC. Palmquist et al. indicated that patients with a high level of CA19-9, interleukin-6, and human cartilage glycoprotein-40 had a shorter OS than patients with lower levels (84). Besides, the study by Gu et al. analyzed 109 Chinese PDAC patients and indicated that the preoperative level of CA19-9 was independently correlated with PDAC OS (85). However, the role of CA19-9 in PDAC post-operative recurrence and prognosis has not been systematically reviewed (86). As far as we know, no systematic reviews or meta-analyses discussed the prognostic value of CA19-9 in PDAC patients. And it is the reason why CA19-9 was not included in the present study. Therefore, the potential of CA19-9 as a potential prognostic biomarker should be comprehensively explored in future studies.

### Limitations

The present umbrella review is the first to make a comprehensive survey about the associations between PDAC survival outcomes and prognostic biomarkers. However, several limitations still exist in our study. Firstly, the methodological quality of the included systematic reviews is generally poor, thus the interpretation of the results in this umbrella review should be questioned. It may be the reason why no strong evidence exists in our study. Besides, some prognostic biomarkers of PDAC including CA19-9 were not comprehensively evaluated by systematic reviews or meta-analyses. Therefore, these biomarkers were not included in this study. Secondly, most of the eligible meta-analyses include <10 studies, which weakened the power of the statistical tests to identify small-study effects and excess significance. Thirdly, diagnostic biomarkers of PDAC were not evaluated in our study which should be further assessed in the future. Fourthly, more than half of the included meta-analyses were conducted in one country (China) rather than evenly distributed around the world which may cause some bias and be unrepresentative.

## Conclusion

The associations between CAR or NLR and PDAC OS were supported by highly suggestive evidence. And the association between LDH and PDAC OS was supported by suggestive evidence. However, these results should be cautiously interpreted due to the relatively inferior methodological quality of the included meta-analyses evaluated by AMSTAR2.0.

## Data Availability Statement

All datasets generated for this study are included in the article/Supplementary Material.

## Author Contributions

JG and YW conceived and designed the study. YW and XZ performed the literature search, and acquired and collated the data, which were analyzed by LZ, JL, BJ and CL. JG was guarantor, and attests that all listed authors meet authorship criteria and that no authors meeting the criteria have been omitted. All authors drafted and critically revised the manuscript for important intellectual content, and gave final approval of the version to be published and contributed to the manuscript.

## Conflict of Interest

The authors declare that the research was conducted in the absence of any commercial or financial relationships that could be construed as a potential conflict of interest.
