# Supplementary material for: Prognostic Biomarkers for Pancreatic Ductal Adenocarcinoma: An Umbrella Review
Source: Front Oncol. 2020 Sep 17;10:1466. doi: 10.3389/fonc.2020.01466 (PMC7527774; doi:10.3389/fonc.2020.01466)
Supplement: Supplementary file 3 [file Table_3.docx]

**Supplementary Table S3.** Results of statistical analyses for the evidence rating of the included systematic reviews and meta-analyses

| **Study** | **Prognosis-indicative role of a biomarker** | **Summary relative risk (random effect)** | | **Cases** | **Largest study** | **I^2^** | ***P* value for Egger’s test** | **95% prediction interval** | **Excess significance test** | | **10% credibility ceiling** |
| --- | --- | --- | --- | --- | --- | --- | --- | --- | --- | --- | --- |
|  |  | **Estimate** | ***P***  **value** | ***N*** | **Relative risk estimate (95% CI)** |  |  |  | ***O/E*** | ***P***  **value** |  |
| **Associations supported by highly suggestive evidence (2)** | | | | | | | | | | | |
| Fu et al. (33) | CAR OS | 1.86  (1.53-2.26) | 5.24*  10^-10^ | 1666 | 2.07  (1.59-2.70) | 54.7% | 0.935 | (1.04-3.30) | 9/4.32 | 0.004 | 2.90*10^-4^ |
| Zhou et al. (59) | NLR OS | 1.81  (1.59-2.05) | 6.75*  10^-20^ | 6457# | 1.09  (1.02-1.16) | 81.7% | <0.001 | (0.90-3.63) | 36/7.08 | 1.31*10^-32^ | 1.33*10^-6^ |
| **Associations supported by suggestive evidence (1)** | | | | | | | | | | | |
| Gan et al. (53) | LDH OS | 1.57  (1.30-1.90) | 2.92*10^-6^ | 3137 | 1.82  (1.38-2.38) | 69.7% | 0.740 | (0.76-3.24) | 11/3.19 | 1.43*10^-6^ | 0.014 |
| **Associations supported by weak evidence (41)** | | | | | | | | | | | |
| Luo et al. (30) | AEG-1 OS | 2.41  (1.63-3.57) | 1.18*10^-5^ | 82# | 2.64  (1.54-4.53) | 0% | - | - | 2/1.61 | 0.49 | 0.072 |
| Chen et al. (31) | B7-H4 OS | 3.00  (2.20-4.10) | 3.77*10^-12^ | 349 | 0.49  (0.27-0.87) | 0% | 0.938 | (1.81-4.97) | 4/1.42 | 0.01 | 0.006 |
| Smith et al. (32) | Bax OS | 0.51  (0.38-0.67) | 1.32*10^-6^ | 185# | 0.56  (0.33-0.95) | 0% | 0.253 | (0.32-0.79) | 5/1.45 | 4.67*10^-4^ | 0.001 |
|  | Bcl-2 OS | 0.51  (0.39-0.68) | 4.80*10^-6^ | 267 | 0.56  (0.33-0.96) | 0% | 0.973 | (0.32-0.82) | 3/0.63 | 1.40*10^-3^ | 5.10*10^-4^ |
|  | P16 OS | 0.63  (0.43-0.92) | 0.017 | 28# | 0.82  (0.50-1.33) | 9.1% | 0.018 | (0.04-10.26) | 3/2.05 | 0.39 | 0.10 |
|  | VEGF OS | 1.51  (1.18-1.92) | 9.75*10^-4^ | 537# | 1.46  (1.02-2.09) | 54.8% | 0.269 | (0.73-3.13) | 5/2.77 | 0.12 | 0.04 |
| Li et al. (34) | CD133 5-year OS | 0.57  (0.48-0.67) | 9.57*10^-12^ | 552# | 0.62  (0.42-0.90) | 9.5% | 0.025 | (0.43-0.74) | 8/2.76 | 2.68*10^-4^ | 3.52*10^-4^ |
| Liu et al. (35) | CD44 5-year OS | 0.51  (0.29-0.92) | 0.024 | 284 | 0.45  (0.15-1.34) | 0% | 0.600 | (0.2-1.32) | 1/0.87 | 0.88 | 0.09 |
| Samarendra et al. (36) | CXCL12s OS | 1.54  (1.21-1.97) | 5.15*10^-4^ | 354 | 1.41  (0.95-2.10) | 0% | 0.102 | (0.90-2.64) | 0/0.48 | 0.46 | 0.01 |
|  | CXCL12s PFS | 1.79  (1.05-3.04) | 0.03 | 84 | 1.95  (1.09-3.48) | 0% | - | - | 1/0.33 | 0.20 | 0.26 |
| Ding et al. (37) | CXCR4 PFS | 1.44  (1.01-2.06) | 0.045 | 1131 | 1.03  (0.73-1.46) | 38.8% | 0.063 | (0.61-3.42) | 1/0.40 | 0.33 | 0.28 |
| Fan et al. (38) | CXCR7 OS | 1.46  (1.12-1.90) | 0.006 | 503 | 1.47  (0.99-2.16) | 0% | 0.576 | (0.26-8.21) | 0/0.51 | 0.43 | 0.053 |
| Wang et al. (39) | COX-2 OS | 1.59  (1.26-2.01) | 8.64*10^-5^ | 498# | 0.78  (0.69-0.89) | 0% | 0.490 | (1.15-2.21) | 2/3.36 | 0.26 | 0.04 |
| Stephenson et al. (40) | DTCs/CTCs OS | 1.84  (1.37-2.45) | 4.16*10^-5^ | 561# | 1.32  (0.99-1.78) | 47.2% | 0.166 | (0.84-3.99) | 4/3.64 | 0.051 | 0.008 |
|  | DTCs/CTCs PFS | 1.93  (1.19-3.11) | 0.007 | 272 | 1.34  (0.94-1.90) | 53.9% | 0.306 | (0.30-12.16) | 2/0.23 | 1.44*10^-4^ | 0.04 |
| Ye et al. (41) | DLL4 OS | 2.13  (1.36-3.33) | 8.81*10^-4^ | 105 | 2.05  (1.13-3.71) | 0% | - | - | 2/0.41 | 0.005 | 0.07 |
|  | Notch3 OS | 2.05  (1.49-2.81) | 1.12*10^-5^ | 53# | 1.95  (1.20-3.19) | 0% | 0.066 | (0.26-16.20) | 3/1.23 | 0.04 | 0.03 |
| Jamieson et al. (42) | E-cadherin OS | 1.57  (1.10-2.24) | 0.012 | 172 | 1.82  (1.17-2.83) | 4.9% | - | - | 1/0.38 | 0.26 | 0.07 |
|  | Ki-67 OS | 2.08  (1.14-3.80) | 0.017 | 196 | 3.92  (2.70-5.69) | 77.0% | 0.425 | (0.15-28.28) | 1/2.45 | 0.14 | 0.004 |
|  | P21 OS | 0.49  (0.26-0.92) | 0.03 | 154 | 0.35  (0.18-0.67) | 57.6% | 0.935 | (6.12*10^-4^-398) | 2/0.45 | 0.01 | 0.25 |
|  | TP OS | 2.03  (1.22-3.38) | 0.006 | 120 | 1.97  (1.16-13.34) | 0% | - | - | 1/0.43 | 0.33 | 0.096 |
| Sharen et al. (45) | Glut1 OS | 1.83  (1.21-2.79) | 0.005 | 451# | 0.84  (0.80-1.20) | 82.2% | 0.001 | (0.46-7.35) | 5/1.39 | 7.56*10^-4^ | 0.07 |
| Bird et al. (47) | hENT1 OS | 0.53  (0.38-0.73) | 1.20*10^-4^ | 683 | 0.60  (0.43-0.83) | 65.0% | 0.187 | (0.20-1.40) | 6/1.04 | 1.36*10^-7^ | 0.048 |
|  | hENT1 DFS | 0.58  (0.42-0.79) | 5.36*10^-4^ | 313# | 0.64  (0.47-0.89) | 54.9% | 0.09 | (0.24-1.40) | 4/1.52 | 0.02 | 0.054 |
| Ye et al. (49) | HIF-1α OS | 1.88  (1.39-2.56) | 4.97*10^-5^ | 262# | 1.94  (1.19-3.17) | 0% | 0.625 | (1.2-2.90) | 2/1.93 | 0.95 | 0.008 |
| Luo et al. (50) | HIF-2α OS | 1.97  (1.42-2.74) | 5.24*10^-5^ | 411 | 1.68  (1.27-2.22) | 23.6% | 0.261 | (0.11-36.16) | 3/0.44 | 2.94*10^-5^ | 0.03 |
| Wu et al. (52) | HMGB1 OS | 2.61  (1.48-4.59) | 9.10*10^-4^ | 101 | 2.49  (1.20-4.80) | 0% | - | - | 2/0.55 | 0.02 | 0.07 |
| Mao et al. (54) | LMR OS | 0.59  (0.41-0.85) | 0.005 | 668 | 0.58  (0.42-0.80) | 68.0% | 0.639 | (0.17-2.02) | 3/0.99 | 0.02 | 0.14 |
| Mehrad-Majd et al. | LncRNA loc285194 OS | 1.97  (1.05-3.68) | 0.03 | 108 | 2.42  (1.21-4.84) | 7.8% | - | - | 1/1.13 | 0.85 | 0.29 |
| Liu et al. (55) | LncRNA UCA1 OS | 1.62  (1.15-2.28) | 0.006 | 160 | 1.50  (1.01-2.24) | 0% | - | - | 2/0.30 | 7.61*10^-4^ | 0.08 |
| Zhou et al. (59) | NLR DFS | 1.65  (1.17-2.35) | 0.005 | 1141 | 1.58  (1.08-2.31) | 67.6% | 0.516 | (0.57-4.82) | 5/1.03 | 2.78*10^-5^ | 0.13 |
| Hu et al. (60) | PD-L1 OS | 1.63  (1.34-1.98) | 1.02*10^-6^ | 766 | 1.58  (1.10-2.27) | 0% | 0.713 | (1.28-2.08) | 3/1.24 | 0.09 | 0.005 |
|  | PD-L1 CSS | 1.86  (1.35-2.57) | 1.75*10^-4^ | 381 | 2.04  (1.26-3.30) | 0% | 0.501 | (0.23-15.22) | 2/1.11 | 0.29 | 0.03 |
| Ji et al. (61) | Plasma fibrinogen OS | 1.56  (1.13-2.16) | 0.007 | 701 | 1.34  (1.03-1.74) | 75.5% | 0.872 | (0.54-4.52) | 5/0.54 | 1.99*10^-10^ | 0.14 |
| Hu et al. (64) | Podoplanin^+^fbroblast OS | 2.20  (1.40-3.46) | 6.73*10^-4^ | 184 | 2.15  (1.12-4.15) | 0% | 0.293 | (0.12-41.57) | 2/0.92 | 0.18 | 0.09 |
|  | Podoplanin^+^fbroblast DFS | 1.97  (1.37-2.84) | 2.51*10^-4^ | 165 | 1.80  (1.12-2.90) | 0% | - | - | 2/0.46 | 0.01 | 0.07 |
| Zhang et al. (66) | RRM1 OS | 1.61  (1.27-2.04) | 8.58*10^-5^ | 666 | 1.74  (1.56-1.90) | 58.5% | 0.605 | (0.88-2.95) | 8/1.06 | 7.15*10^-13^ | 0.006 |
| Wang et al. (67) | Smad4 OS | 0.61  (0.37-0.99) | 0.046 | 1446# | 0.62  (0.43-0.89) | 67.5% | 0.599 | (0.14-2.59) | 4/3.72 | 0.85 | 0.04 |
| Han et al. (68) | SPARC OS | 1.55  (1.11-2.18) | 0.01 | 908# | 1.02  (0.73-1.43) | 73.1% | 0.117 | (0.53-4.54) | 4/1.30 | 0.009 | 0.21 |
| Wu et al. (69) | STAT3 5-year OS | 9.72  (1.80-52.45) | 0.008 | 17 | 7.85  (0.43-144.32) | 0% | 0.278 | (1.74*10^-4^-5.42*10^5^) | 0/2.19 | 0.004 | 0.03 |
| Chen et al. (70) | ZEB1 OS | 1.49  (1.07-2.07) | 0.017 | 120 | 1.42  (0.97-2.19) | 0% | - | - | 0/0.28 | 0.57 | 0.07 |
| **Associations supported by not suggestive evidence (19)** | | | | | | | | | | | |
| Smith et al. (32) | EGFR OS | 1.35  (0.80-2.27) | 0.26 | 178# | 0.79  (0.47-1.34) | 58.6% | 0.298 | (0.17-10.79) | 1/1.18 | 0.84 | 0.57 |
|  | P53 OS | 1.22  (0.96-1.56) | 0.10 | 550 | 0.90  (0.64-1.26) | 57.6% | 0.298 | (0.53-2.81) | 5/4.68 | 0.86 | 0.98 |
| Liu et al. (43) | FGFR2 3-year OS | 1.65  (0.41-6.55) | 0.48 | 135 | 2.83  (1.06-7.60) | 66.1% | 0.766 | (3.32*10^-7^-8.18*10^6^) | 1/1.27 | 0.75 | 0.005 |
|  | FGFR2 5-year OS | 1.05  (0.38-2.91) | 0.93 | 148 | 1.60  (0.58-4.41) | 17.6% | 0.372 | (2.22*10^-4^-4938) | 0/0.33 | 0.54 | 0.90 |
| Dai et al. (44) | FoxM1 OS | 1.91  (0.92-3.94) | 0.08 | 237 | 1.27  (0.71-2.83) | 47.2% | 0.221 | (0.001-2862) | 1/0.20 | 0.06 | 0.16 |
| Cao et al. (46) | HDAC1 OS | 1.43  (0.71-2.88) | 0.32 | 208 | 1.02  (0.60-1.75) | 73.4% | 0.150 | (0.07-29.62) | 1/0.20 | 0.07 | 0.87 |
| Li et al. (48) | HER2 OS | 1.87  (0.64-5.46) | 0.25 | 132# | 0.79  (0.44-1.41) | 86.8% | 0.055 | (0.01-257) | 2/2.11 | 0.91 | 0.75 |
| Wu et al. (51) | HK2 OS | 1.55  (0.50-4.85) | 0.45 | 110# | 2.70  (1.76-4.15) | 91.0% | 0.939 | (1.25*10^-6^-1.93*10^6^) | 1/1.10 | 0.90 | 0.57 |
| Hua et al. (57) | LICAM OS | 0.96  (0.41-2.22) | 0.92 | 257 | 0.95  (0.57-1.58) | 85.7% | 0.533 | (3.34*10^-5^-2.76*10^4^) | 1/0.15 | 0.02 | 0.96 |
| Shao et al. (58) | MiRNA-203 OS | 1.29  (0.97-1.73) | 0.08 | 142# | 1.18  (1.08-1.30) | 38.5% | 0.455 | (0.07-23.47) | 2/1.11 | 0.29 | 0.096 |
| Jamieson et al. (42) | pAkt OS | 0.83  (0.05-12.92) | 0.90 | 74 | 0.21  (0.10-0.45) | 94.9% | - | - | 2/1.33 | 0.32 | 0.96 |
|  | P27 OS | 0.96  (0.49-1.88) | 0.91 | 309 | 1.28  (0.82-1.99) | 79.7% | 0.468 | (0.09-10.33) | 3/0.33 | 1.51*10^-6^ | 0.58 |
|  | Survivin OS | 0.28  (0.06-1.35) | 0.11 | 105 | 0.56  (0.34-0.91) | 80.5% | - | - | 2/0.19 | 1.27*10^-5^ | 0.12 |
|  | TS OS | 0.91  (0.27-3.06) | 0.88 | 176 | 1.66  (1.05-2.63) | 90.2% | - | - | 2/0.32 | 0.001 | 0.94 |
| Zhou et al. (62) | PLR OS | 1.00  (0.92-1.09) | 0.93 | 591 | 0.98  (0.90-1.08) | 0% | 0.207 | (0.59-1.72) | 0/0.15 | 0.69 | 0.93 |
| Zhu et al. (63) | PKM2 OS | 1.41  (0.68-2.93) | 0.36 | 259 | 0.57  (0.36-0.91) | 81.3% | 0.430 | (0.05-36.54) | 2/0.66 | 0.07 | 0.36 |
| Yu et al. (65) | RKIP OS | 0.79  (0.58-1.07) | 0.13 | 256 | 0.95  (0.68-1.34) | 30.6% | 0.404 | (0.05-12.44) | 1/0.15 | 0.02 | 0.29 |
|  | RKIP DFS | 0.69 (0.36-1.31) | 0.26 | 237 | 1.16  (0.86-1.56) | 80.3% | 0.147 | (3.51*10^-4^-1356) | 2/0.17 | 4.88*10^-6^ | 0.50 |
| Zhang et al. (66) | RRM1 DFS | 1.47  (0.83-2.62) | 0.19 | 185 | 1.80  (1.55-2.22) | 70.8% | 0.552 | (0.002-927) | 3/0.47 | 5.86*10^-5^ | 0.59 |

-: Not available

**Abbreviations**: CI: confidence interval; OS: overall survival; CSS: cancer-specific survival; DFS: disease free survival; PFS: progression free survival ; AEG-1:Astrocyte elevated gene-1; CAR: C-reactive protein to albumin ratio; COX-2: Cyclooxygenase-2; DLL4:Delta like ligand 4; DTCs/CTCs: Disseminated tumor cells/ circulating tumor cells; EGFR: Epidermal growth factor receptor; VEGF: Vascular endothelial growth factor; FGFR2: Fibroblast growth factor receptors; FoxM1:Forkhead Box M1; GLUT1: Glucose transporter type 1; HDAC1:Histone deacetylase 1; hENT1: human equilibrative nucleoside transporter 1; HER2: Human epidermal growth factor receptor-2; HIF-1α: Hypoxia inducible factor-1α; HIF-2α:Hypoxia inducible factor-2α; HK2: Hexokinase 2; HMGB1: High mobility group box 1; LDH: Lactate dehydrogenase; LMR: Lymphocyte-to-monocyte ratio; LncRNA: long non-coding RNA; L1CAM: L1 cell adhesion molecule; MiRNA: MicroRNA; NLR: Neutrophil-to-lymphocyte ratio; pAkt: Phosphorylated protein kinase B; PD-L1: Programmed cell Death Ligand 1; PLR : Platelet-to-lymphocyte ratio; PKM2: Pyruvate kinase M2; RKIP: Raf kinase inhibitor protein; RRM1: Ribonucleotide reductase M1; SPARC: Secreted protein acidic and rich in cysteine; STAT3: Signal transducer and activator of transcription proteins 3; TS: Thymidylate synthase; TP: Thymidylate phosphorylase; VEGF: Vascular endothelial growth factor; ZEB1:Zinc finger E-box binding homeobox 1.
